# Compound Acoustic Radiation Force Impulse Imaging of Bovine Eye by Using Phase-Inverted Ultrasound Transducer

**DOI:** 10.3390/s24092700

**Published:** 2024-04-24

**Authors:** Gil Su Kim, Hak Hyun Moon, Hee Su Lee, Jong Seob Jeong

**Affiliations:** Department of Biomedical Engineering, Dongguk University, Seoul 04620, Republic of Korea; kimpak85@dongguk.edu (G.S.K.); moonhak@dongguk.edu (H.H.M.); 22sooooo@dongguk.edu (H.S.L.)

**Keywords:** compound acoustic radiation force impulse imaging, phase-inverted ultrasound transducer, internal ocular structure, depth-of-field

## Abstract

In general, it is difficult to visualize internal ocular structure and detect a lesion such as a cataract or glaucoma using the current ultrasound brightness-mode (B-mode) imaging. This is because the internal structure of the eye is rich in moisture, resulting in a lack of contrast between tissues in the B-mode image, and the penetration depth is low due to the attenuation of the ultrasound wave. In this study, the entire internal ocular structure of a bovine eye was visualized in an ex vivo environment using the compound acoustic radiation force impulse (CARFI) imaging scheme based on the phase-inverted ultrasound transducer (PIUT). In the proposed method, the aperture of the PIUT is divided into four sections, and the PIUT is driven by the out-of-phase input signal capable of generating split-focusing at the same time. Subsequently, the compound imaging technique was employed to increase signal-to-noise ratio (SNR) and to reduce displacement error. The experimental results demonstrated that the proposed technique could provide an acoustic radiation force impulse (ARFI) image of the bovine eye with a broader depth-of-field (DOF) and about 80% increased SNR compared to the conventional ARFI image obtained using the in-phase input signal. Therefore, the proposed technique can be one of the useful techniques capable of providing the image of the entire ocular structure to diagnose various eye diseases.

## 1. Introduction

It is well known that each ocular tissue has individual biomechanical properties, and an eye is known as a complex organ with a close relationship between its dysfunction and change in biomechanical properties [1,2,3,4,5,6]. For example, keratoconus and presbyopia are common ocular diseases in a cornea and a crystalline lens. The keratoconus caused by abnormal corneal collagen-fibril mesh leads to a change in corneal stiffness [7]. Presbyopia is an age-related disease where the control ability of the crystalline lens decreases, and it is caused by the gradual hardening of the crystalline lens due to aging [8]. The cataract is a disease in which the crystalline lens becomes cloudy and similar to the presbyopia in that most cataracts are age-related cataracts (senile cataracts) and affect the stiffness of the crystalline lens [9,10]. Although the cataract develops secondary to other eye diseases or accelerates in certain systemic conditions, if the senile cataract occurs in the presence of the presbyopia, the elasticity of the crystalline lens further decreases, worsening the presbyopia [11,12]. Therefore, determining the extent of the cataract progression using accurate assessment of the condition of the lens plays an important role in determining the timing and whether to perform the cataract surgery.

Currently, the most popular clinical method used to assess the degree of the cataract is optical coherence tomography (OCT), and the OCT can mainly provide information on morphological change in the crystalline lens rather than biomechanical alteration of the crystalline lens. As several studies have shown that cataracts accompany biomechanical property changes that increase the stiffness of the lens, monitoring the biomechanical properties of the lens emerges as important information that can be helpful in diagnosing cataracts [13].

Various methods have been proposed to measure the changes in the biomechanical properties of the crystalline lens, and elasticity-based imaging techniques have been developed to monitor alterations in noninvasive ways instead of some invasive techniques with clinical feasibility limitations [14,15]. The elasticity is a property in which the tissue tries to return to its original form when the external force that induced deformation is removed, and the tissue elasticity imaging visualizes the difference in stiffness between targets by detecting the tissue elasticity [16,17]. Elastography, one of the tissue elasticity imaging methods, applies compression to the tissue using external mechanical excitation and then applies the correlation algorithm between pre-compression and post-compression echo signals for estimating the tissue strain or displacement [16,17]. Several imaging modalities, such as ultrasound, OCT, and magnetic resonance imaging (MRI), were used to detect the tissue motion. However, in the ophthalmology field that requires a high spatial resolution, optical coherence elastography (OCE) and ultrasound elastography (USE) are more suitable than magnetic resonance elastography (MRE) with a low spatial resolution and long image acquisition time [18,19].

The application of OCE in the ophthalmic field especially has advantages in resolution and transparency [20,21]. The acoustic radiation force optical coherence elastography (ARF-OCE) [1,22,23] method that uses the acoustic radiation force by ultrasound as an excitation force also provides better resolution than acoustic radiation force-based ultrasound elastography (ARF-USE) [2,24,25]. However, the ARF-OCE has the problem of lower penetration depth compared to the ARF-USE in observing the lens located deeper than the cornea, among other ocular structures [24,25]. Additionally, the ARF-USE has the advantage over the ARF-OCE in that it does not require focal alignment between the ultrasound transducer for stimulation and the OCT for detection, and the elastic information can be obtained only with the ultrasound transducer.

Among the several ARF-USE techniques, the acoustic radiation force impulse (ARFI) imaging estimates the localized axial displacement quantitatively generated using the impulsive acoustic radiation force. Note that the magnitude of the estimated displacement is inversely proportional to the tissue stiffness [26,27,28,29,30]. The ARFI image has been widely applied to several human organs, such as the breast, liver, and prostate, because it can provide mechanical properties of the object that B-mode imaging cannot provide [31,32,33]. The beam sequence of the ARFI imaging consists of a reference beam and tracking beams to monitor tissue location and a pushing beam to induce tissue deformation. In other words, ARFI imaging is a strain imaging method that transmits the tracking beam at the exact location using the same transducer at regular intervals after transmitting the pushing beam. However, in the displacement calculation using the pushing beam and the tracking beams in the same transducer, a decorrelation called a shearing artifact, which is an echo decorrelation between the reference beam and the tracking beam, occurs. The shearing artifact is a phenomenon in which the on-axis scatter distribution within the point spread function (PSF) of the tracking beam becomes non-uniform due to the gradient of force generated by the pushing beam when the beamwidth of the tracking and pushing beams were fixed the same [34,35,36]. Due to the increased variance, the finally calculated displacement does not sufficiently represent the actual tissue movement, and the increased jitter and the peak displacement underestimation caused by the shearing artifact reduce the resolution of the ARFI image [37,38,39]. To solve this issue, several researchers have shown that a broadly defocused pushing beam and a narrowly focused tracking beam enable high correlation and accurate displacement estimation with reduced shearing artifacts in ARFI images [36,39].

Based on this research, the array transducer and the dual-element transducer were used to obtain the relatively broad pushing beam [6,34,40,41,42]. In the case of the array transducer, the beamwidth can be effectively controlled, but compared to the case of using a single-element transducer, it is not easy to manufacture, and the system is complicated for the individual driving of each array element. A dual-element transducer has the advantage of selectively transmitting a different center frequency for each element and adjusting the beamwidth of the pushing beam to be broader than the tracking beam. However, it has the disadvantage that it is challenging to align between two elements accurately.

To solve this problem, in our previous study, we proposed the phase-inverted ultrasound transducer (PIUT) to increase the beamwidth of the pushing beam relative to the tracking beam [43]. The performance was demonstrated via experiments with a prototype transducer and a tissue-mimicking phantom. In the same vein, in this paper, we demonstrated the effect of the PIUT on improving ARFI image quality by applying it to an ex vivo environment, i.e., a bovine eye. Additionally, in order to improve the quality of the ARFI image by increasing the signal-to-noise ratio (SNR) and by reducing displacement error, the compound ARFI (CARFI) images were obtained by combining several mode images acquired via repeated experiments. The proposed method can provide an excellent ARFI image of the entire internal structure of the eye, and about 80% increased SNR compared to the conventional ARFI image. Thus, it can be useful in diagnosing and treating various eye diseases, not only in the anterior segment but also in the posterior segment. Section 2 describes the working principle of PIUT, its fabrication process, and the experimental setup. Section 3 illustrates the experimental results using the bovine eye and prototype PIUT. Discussion and Conclusion on the proposed technique are illustrated in Section 4 and Section 5, respectively.

## 2. Materials and Methods

### 2.1. Principle and Simulation of Phase-Inverted Ultrasound Transducer

In the field of therapeutic ultrasound, the split-focus technique can shorten treatment time because it can expand the coagulated area with each ultrasound sonication [44,45,46]. To achieve split-focusing, the phase-inverted ultrasonic transducer (PIUT) with a four-segment aperture driven by a phase-inverted input signal is proposed [43]. In the in-phase mode, the input signals of the same phase (0°) are applied to the transducer to produce a single focus. In the out-of-phase mode, the input signals are transmitted with a 180° phase difference to each adjacent element to create four foci simultaneously. Employing this technique for ARFI imaging can alleviate the shearing artifact by increasing the lateral beamwidth of the pushing beam and can also extend a depth-of-field (DOF) [43,47,48,49,50].

**Figure 1:**
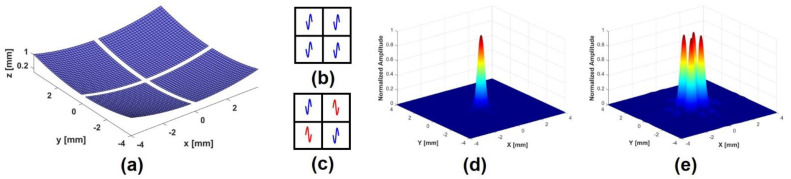
Field II simulation results; (**a**) aperture of PIUT, (**b**) in–phase mode input signal, (**c**) out–of–phase mode input signal, (**d**) 3D ultrasound intensity at focal point in in–phase mode, (**e**) 3D ultrasound intensity at focal point in out–of–phase mode. Note that the blue/red lines in (**b**,**c**) represent sinusoidal input signals, and the colors in (**d**,**e**) represent normalized amplitude.

**Figure 2:**
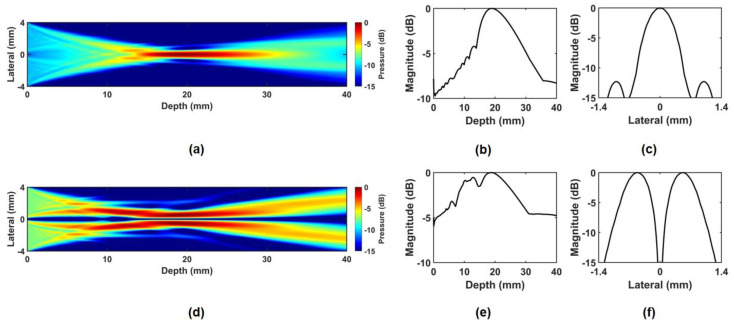
FEA simulation results; (**a**) 2D beam profile of in–phase mode, (**b**) axial beam plot of in–phase mode, (**c**) lateral beam plot of in–phase mode, (**d**) 2D beam profile of out–of–phase mode, (**e**) axial beam plot of out–of–phase mode, (**f**) lateral beam plot of out–of–phase mode.

To demonstrate the performance of the proposed method, two kinds of ultrasound field simulations were performed using the Field-II program and the finite element analysis (FEA) based program (OnScale, Atlanta, GA, USA), which are widely used in various ultrasound simulations [43,51,52]. The Field-II program can provide relative acoustic profiles, and the FEA simulation can provide absolute acoustic profiles. In particular, since the FEA simulation is performed based on the properties of materials actually used, it can provide the design parameters necessary for fabricating a prototype transducer. The center frequency of the PIUT was determined to be 5 MHz, which was the highest center frequency for our hardware environment, considering the target size, input sequence composition for ARFI imaging, and the sampling frequency for the data acquisition (DAQ) board (CS121G2, Vitrek Corp., Poway, CA, USA) driven by LabVIEW program (National Instruments Corp., Austin, TX, USA). The aperture size was 8.1 mm × 8.1 mm, the center kerf was 0.1 mm, and the focal depth was determined to be 20 mm, considering the size of the ocular target. In the Field II simulation, the four-segment aperture was designed, and subsequently, the 3D intensity plots were obtained, as shown in Figure 1. Figure 1a is the schematic diagram of the simulated aperture of PIUT. Figure 1b,c show the phases of the applied input signals. For the in-phase mode, the same phase signals are used (Figure 1b), and for the out-of-phase mode, the phase-inverted signals are used (Figure 1c). Figure 1d,e show three-dimensional (3D) normalized intensity at the focal point. In the in-phase mode, a single focal point was generated, while four focal points were produced in the out-of-phase mode. This phenomenon can also be verified in the FEA simulation, as shown in Figure 2. Due to hardware limitations and simulation time, two-dimensional (2D) simulation was performed instead of 3D simulation. However, the accuracy of the 2D simulation is sufficiently high compared to the C-mode pressure fields obtained experimentally. To reduce reflected ultrasound waves returning from the medium boundary, all boundary conditions of the water medium were set to an absorption layer. In the 2D beam profile of the in-phase mode, the single focal point can be observed, as shown in Figure 2a. In the 2D beam profile of the out-of-phase mode, two focal points are shown (Figure 2d). Figure 2b,e show the axial beam plots of the in-phase and out-of-phase modes, and it was verified that the −3 dB DOF of the out-of-phase mode was increased by 60%. In the case of lateral beam plot, the out-of-phase mode increases the −3 dB lateral beamwidth by 159% compared to the in-phase mode. Note that the mesh size for all FEA simulations was determined using 25 elements per wavelength for water, which has the smallest wavelength among the materials in the model, taking into account computational time and time resolution.

### 2.2. Transducer Fabrication and Experimental Setup

**Figure 3:**
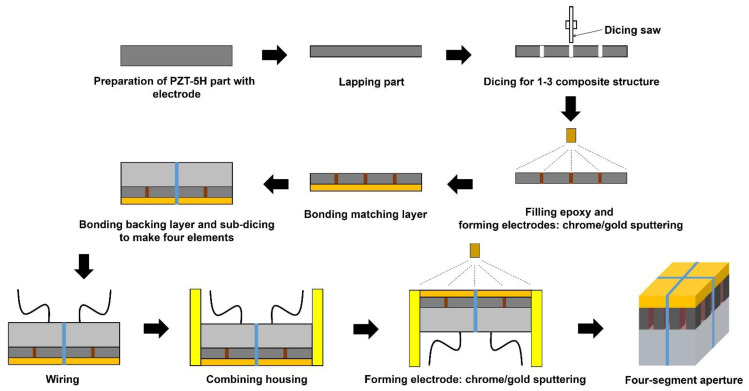
Fabrication process of the prototype PIUT.

The prototype PIUT was newly fabricated, and the simple fabrication process for the PIUT is described in Figure 3. Note that the design specifications of the fabricated prototype transducer are identical to our previous work [53]. First, a bulk-type PZT-5H plate was prepared and lapped for 5 MHz resonance. 1-3 composite structure was formed using a dicing saw (DAD322, DISCO Corp., Tokyo, Japan), and kerfs were filled using unloaded epoxy (Epoxy Technology Inc., Billerica, MA, USA). Chrome/Gold (500 Å/2000 Å) sputtering was conducted to make the electrodes. A matching layer made of silver powder (Aldrich Chem. Co., Milwaukee, WI, USA) and unloaded epoxy (Insulcast 502, ITW Polymer Technologies, Montgomeryville, PA, USA) was bonded to the piezoelectric layer.

**Figure 4:**
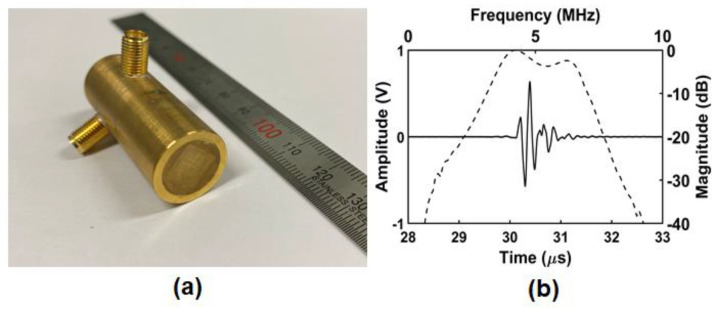
Photograph of (**a**) prototype PIUT and (**b**) measured pulse–echo data.

**Figure 5:**
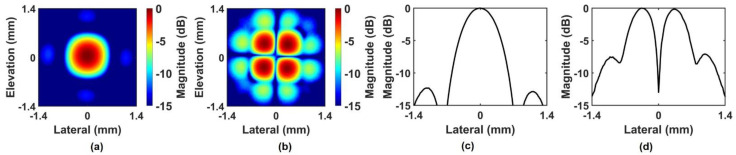
Measured pressure fields in the C–mode and the lateral beam plots: (**a**) C–mode pressure fields for (**a**) in–phase mode and (**b**) out–of–phase mode; Lateral beam plots for (**c**) in–phase mode and (**d**) out–of–phase mode.

A backing layer was cast on the part, and sub-dicing was performed to separate elements. After wiring, it was combined with housing, and the electrode was formed on the aperture using Chrome/Gold (500 Å/2000 Å). Two diagonally positioned elements are connected to each other. Figure 4a shows the photograph of the prototype PIUT, and the four-segment aperture can be seen clearly. Figure 4b shows the pulse-echo experimental result. The center frequency was 5.09 MHz, and the −6 dB fractional bandwidth was 66.41%. Figure 5 shows the measured pressure fields in the constant depth mode (C-mode) and the lateral beam plots. Figure 5a,b show the C-mode pressure fields for the in-phase mode and the out-of-phase mode, respectively. Figure 5c,d show the lateral beam plots for the in-phase mode and the out-of-phase mode, respectively, based on one-way propagation. The −3 dB lateral beamwidths were 0.71 mm and 1.2 mm in the in-phase and the out-of-phase modes, respectively. That is, the −3 dB lateral beamwidth of the out-of-phase mode was improved by 69% when the center valley was included. In the FEA simulation, the −3 dB lateral beamwidths of the in-phase mode (Figure 2c) and the out-of-phase mode (Figure 2f) were 0.62 mm and 1.64 mm, respectively. In the C-mode experiments, the −3 dB lateral beamwidths of the in-phase mode (Figure 5c) and the out-of-phase mode (Figure 5d) were 0.71 mm and 1.2 mm, respectively. There are some differences between FEA simulation and experiment; however, the errors are not significant. Additionally, these errors can occur due to limitations in 2D simulation and manufacturing errors in the prototype transducer.

**Figure 6:**
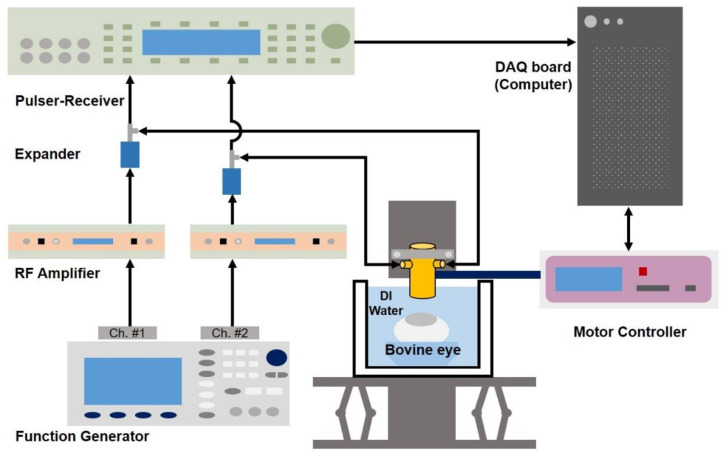
Schematic diagram of the experimental setup using the PIUT.

Figure 6 shows a schematic diagram of the experimental setup for B-mode and ARFI imaging of a bovine eye.

**Figure 7:**
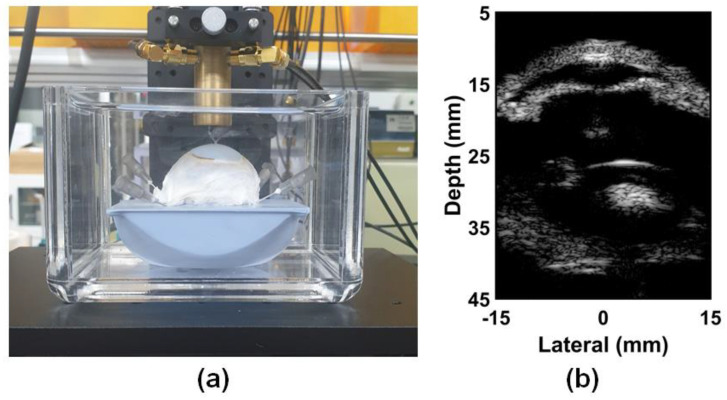
Photograph of (**a**) bovine eye target, and (**b**) obtained B–mode image of (**a**).

Two channels of the function generator (33600A, Keysight Technologies, Santa Clara, CA, USA) are connected to two radio frequency (RF) power amplifiers (75A250A, Amplifier Research, Souderton, PA, USA). The amplified signal was applied to the PIUT, and the received signal was amplified using a pulser-receiver (UT340, UTEX Scientific Instruments Inc., Mississauga, ON, Canada). The target was connected to the linear motor system (SHOT-304GS, SIGMA KOKI, Tokyo, Japan) and controlled using the LabVIEW program. The received RF data were saved on the DAQ board and also controlled using the LabVIEW program. Figure 7a is a photo of the bovine eye target purchased from a laboratory materials sales company (BIOZOA Biologial Supply, Seoul, Republic of Korea). Figure 7b is the B-mode image of the bovine eye, and the B-mode image was obtained using MATLAB (R2022a, The MathWorks, Natick, MA, USA) software. It was logarithmically compressed with a dynamic range of 40 dB. In this B-mode image, cornea, iris, crystalline lens, and retina can be seen.

**Figure 8:**
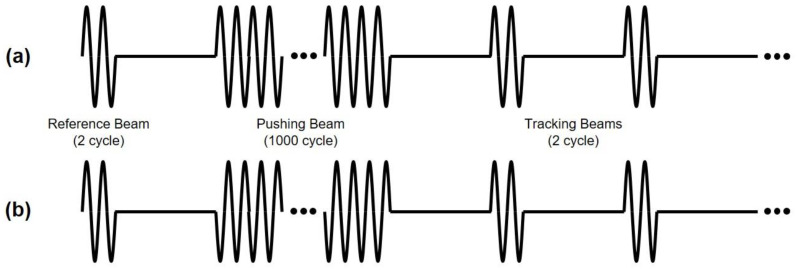
Schematic diagram of the input sequence composed of reference beam, pushing beam, and tracking beams for (**a**) in–phase mode and (**b**) out–of–phase mode. Note that the phases of the pushing beams in the two modes are different.

Generally, in the ultrasound B-mode image, since the acoustic impedance difference between the water-rich vitreous body and the crystalline lens is small, the lens is not clearly visible. However, in this experiment, a bovine eye with cataract symptoms was selected, and thus, the inside of the lens appears partially white in the B-mode image. The position of the lens sank slightly due to gravity. To implement the ARFI image, two types of beam sequences for the in-phase and the out-of-phase modes are described in Figure 8. Both beam sequences are composed of a reference beam with a 2-cycle 5 MHz sine wave, multiple tracking beams with a 2-cycle 5 MHz sine wave, and a pushing beam with a 1000-cycle 5 MHz sine wave. The pulse repetition frequency (PRF) of the multiple tracking beams was set to 10 KHz, considering the target distance. The phases of the pushing beams are different in the in-phase and out-of-phase modes, but the phases of other beam sequences are the same. The output power was adjusted considering the maximum pressure at the focal point is different between the in-phase and out-of-phase modes. Four case data were obtained for each mode.

## 3. Experimental Results

### 3.1. ARFI Imaging of Bovine Eye

**Figure 9:**
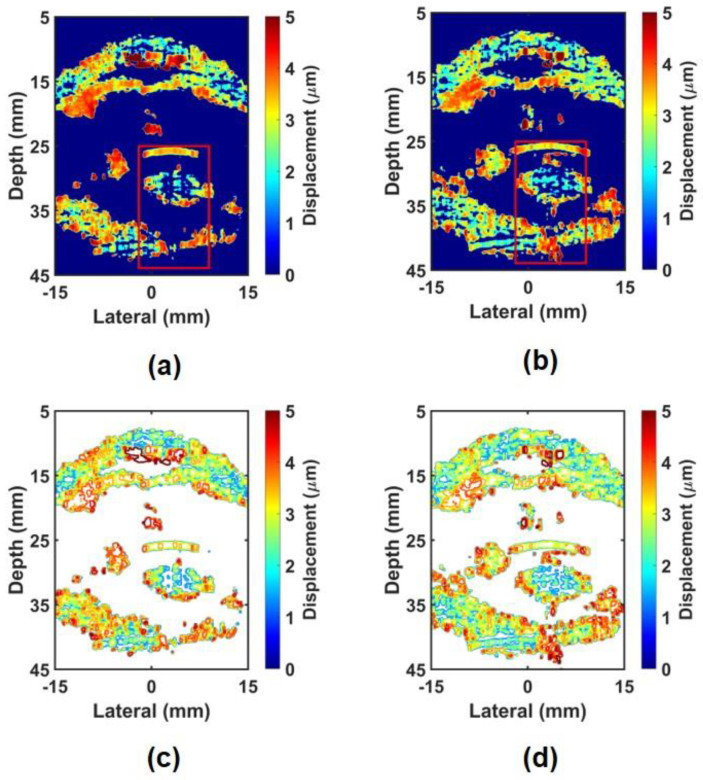
Experimentally obtained non–compound ARFI images and contour plots for bovine eye: ARFI images of (**a**) in–phase mode and (**b**) out–of–phase mode; Contour plots of (**c**) in–phase mode and (**d**) out–of–phase mode.

**Figure 10:**
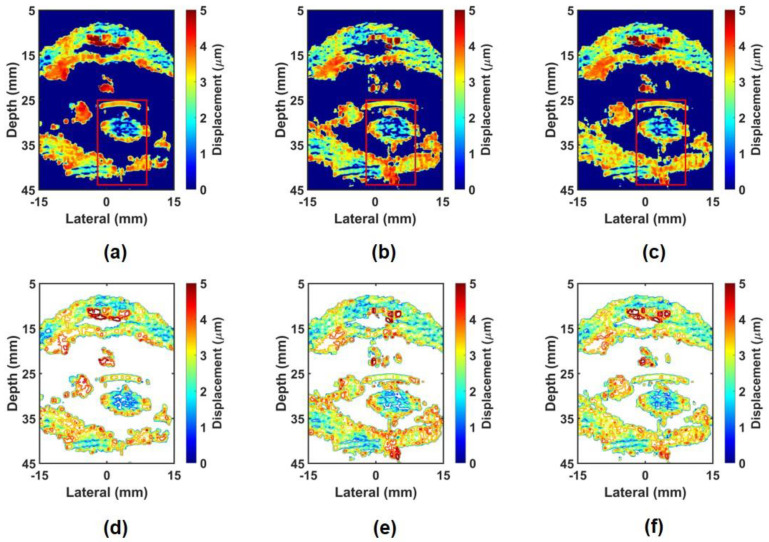
Experimentally obtained CARFI images and contour plots for bovine eye: CARFI images of (**a**) in–phase mode, (**b**) out–of–phase mode, and (**c**) mixed mode (in–phase + out–of–phase); Contour plots of (**d**) in–phase mode, (**e**) out–of–phase mode, and (**f**) mixed mode (in–phase + out–of–phase).

Figure 9 shows the non-compound ARFI images and their contour plots of the in-phase and out-of-phase modes. Figure 9a shows the ARFI image for the in-phase mode, where the middle field shows higher displacement compared to other regions. In the near field, displacement points that are too high are visible due to impulse noise. On the inside of the crystalline lens, a very sparse texture can be seen, and the far-field area shows a very small texture, making it impossible to obtain stiffness information about the target. This trend can be clearly seen in the contour plot shown in Figure 9c. This phenomenon can be explained by the large shearing artifact and the short DOF. On the other hand, Figure 9b,d show the ARFI image and the contour plot for the out-of-phase mode and show a dense texture not only in the near-field area but also in the far-field area, which can provide more precise stiffness information of the target. In particular, the texture inside the crystalline lens is denser than Figure 9a, making it more useful in measuring the stiffness of the target. Figure 10 shows the CARFI images and contour plots using the in-phase mode data and the out-of-phase mode data. When the in-phase mode data were compounded, as shown in Figure 10a,d, the sparse texture was changed to a dense texture compared to the non-compound ARFI image with the in-phase mode (Figure 9a). However, it still shows the sparse texture in the far-field region. Combining the out-of-phase mode data, as shown in Figure 10b,e, not only increased texture density where the texture density was sparse but also increased the texture density in the far field. When the in-phase mode data and out-of-phase mode data are combined (Figure 10c,f), the texture density becomes denser over the entire image, and thus, the far-field area becomes more visible. Using this method, the advantages of in-phase and out-of-phase modes can be taken, and the disadvantages can be compensated.

### 3.2. Performance Evaluation

**Figure 11:**
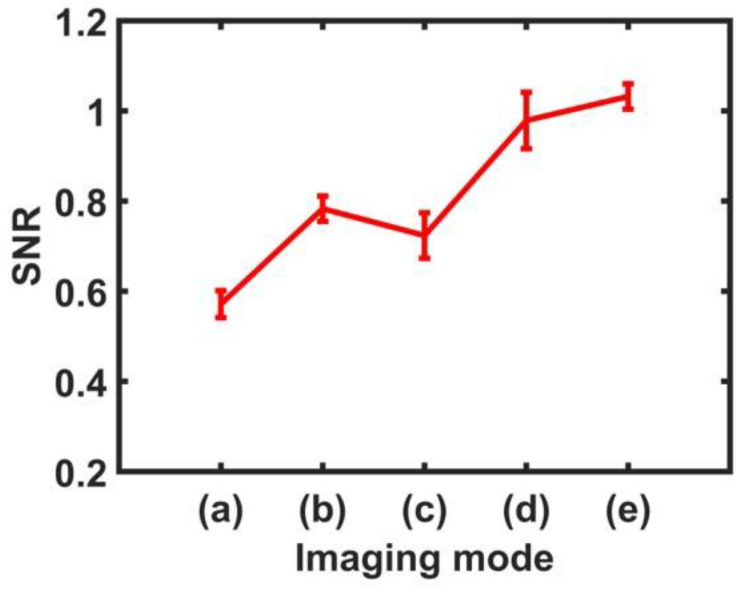
Graphical representation of Table 1. The labels for imaging modes (a–e) correspond to those in Table 1.

Next, for the quantitative performance evaluation of the proposed technique, the SNR calculation within the specified region of interest (ROI) was performed using the following equation

(1) $${SNR}_{ARFI}=\frac{\mu }{\sigma }$$

where *μ* and *σ* is the mean and standard deviation of the estimated displacement, respectively [54,55,56]. The ROI shown in Figure 9 and Figure 10 has a relatively long rectangular shape in the depth direction to confirm the effectiveness of the proposed technique on the displacement representation of the far field and is applied to the identical location of all ARFI images. Thus, the ROI in the red box contains the cataract area within the crystalline lens as well as part of the retina. Table 1 summarizes the SNR calculation results with and without compounding the in-phase and out-of-phase modes. Since four case data were obtained for each mode, the mean SNR was calculated for each mode. Additionally, Figure 11 is a graphical representation of Table 1. Comparing the non-compound ARFI images, it can be seen that the mean SNR values for in-phase and out-of-phase modes are 0.572 and 0.783, respectively. Thus, the SNR of the out-of-phase mode is 36.88% higher than the in-phase mode. On the other hand, CARFI images have mean SNR values of 0.724 in the in-phase mode, 0.979 in the out-of-phase mode, and 1.032 in mixed in-phase and out-of-phase modes. Note that the SNR value can be less than one if the standard deviation of the target displacement is greater than the average value of the target displacement. The SNR improvement values compared to the non-compound ARFI image in the in-phase mode are 26.57% in the CARFI image (in-phase mode), 71.15% in the CARFI image (out-of-phase mode), and 80.42% in CARFI image (mixed in-phase mode and out-of-phase mode).

## 4. Discussion

In general, when implementing the ARFI image, it is important to alleviate the shearing artifact to increase the accuracy of the target displacement calculation. Expanding the lateral beamwidth of the pushing beam reduces the shearing artifact and the jitter, resulting in a high-resolution ARFI image. The proposed PIUT can make the lateral beamwidth of the pushing beam more expansive than the tracking beam by simply adjusting the phase of the applied signal. This feature was demonstrated using both Field II and FEA simulations as well as experiments in this study. In the PIUT consisting of four elements, a single focus is generated in the in-phase mode used for the tracking beam, and four foci are generated in the out-of-phase mode used for the pushing beam. The ARFI images acquired in the in-phase and out-of-phase modes provide the same anatomical information as the B-mode image of the bovine eye and also provide stiffness information of the entire internal structure of the eye, including the crystalline lens with the cataract. Note that this stiffness information cannot be provided by the B-mode image.

The cataract is an ocular disease that causes increased stiffness and opacity of the crystalline lens. In the ARFI image obtained in this experiment, the cataract area with small displacement was colored blue, making it possible to distinguish the cataract area within the crystalline lens. However, the in-phase mode did not offer sufficient stiffness information over the entire region, especially for the deeper region, due to the large shearing artifact and the short DOF. On the contrary, the out-of-phase mode can provide more stiffness information over the entire region and the deeper region that was not available in the in-phase mode. In particular, it can provide biomechanical properties for cataracts based on the reliable stiffness information in the crystalline lens part. For the fair performance comparison, the voltages of the input signals were compensated, and thus, the calculated mean displacement of both modes was similar.

Furthermore, to enhance the quality of the ARFI image, the CARFI scheme was applied by using the ARFI images from the in-phase and out-of-phase modes acquired via repeated experiments. The CARFI images provided a more precise depiction of the target morphology in comparison to the non-compound image from each mode. Thus, the proposed technique can implement a high-resolution ARFI image in terms of distinguishing elastic information between targets and identifying lesion areas that cannot be observed in the B-mode image. Subsequently, to quantitatively analyze single ARFI images and CARFI images in each mode, we assessed the SNR in the ROI positioned to include the surface of the crystalline lens, the cataract region, and the retina. First, comparing the single ARFI images in Figure 9a,b, the out-of-phase mode shows a 36.88% improved mean SNR over the in-phase mode. This is due to the reduced shearing artifact and jitter in the out-of-phase mode, allowing for more accurate displacement calculations and increased DOF.

Comparing CARFI images as shown in Figure 10, the compound image in the out-of-phase mode shows an improved mean SNR value than the in-phase mode. CARFI images outperformed non-compound ARFI images in all modes. As shown in Figure 10c, for the CARFI image in the mixed mode (in-phase + out-of-phase), the mean SNR value was 1.032, showing the highest SNR among all ARFI images. This is because we can obtain both the advantage of the in-phase mode, which provides high displacement in the middle field region and the advantage of the out-of-phase mode, which provides high displacement in the far-field region.

In this paper, SNR was calculated instead of CNR (Contrast to Noise Ratio) to compare the performance of the proposed technique. Due to the characteristics of ocular structures, it is difficult to select an appropriate background region to evaluate CNR [28]. In other words, if the transparent area of the vitreous body is selected as the background area, the average displacement and displacement standard deviation of the background area have values close to zero because they are rich in moisture and have almost no scatterers. In this case, the CNR equation converges to the SNR equation, and thus, the SNR was employed instead of CNR in this study [54,55,56,57,58,59].

In this study, the FEA simulation for the PIUT was performed in the 2D mode rather than the 3D mode due to hardware limitations; however, the simulation results were very similar to the experimental results. Additionally, the center frequency of the transducer was set to 5 MHz, but this frequency has low spatial resolution and is not suitable for real eye monitoring. In general, a higher center frequency increases the spatial resolution but decreases the penetration depth. This phenomenon causes the conventional in-phase mode ARFI eye image to have a shorter DOF. However, using the proposed method, even if the frequency increases, the DOF can be relatively longer than in the general case, making it a suitable technique for eye monitoring. It is well known that diseases such as glaucoma and age-related macular degeneration (AMD) are accompanied by changes in stiffness of the entire posterior ocular tissue connected by the sclera, choroid, and retina rather than specific tissues [60,61,62,63]. Therefore, the possibility of the proposed technique to provide high-resolution elastography of deep regions could be an important advantage in the observation of ocular diseases in the posterior segment, such as the retina and fovea [62,63]. Additionally, the proposed method can be applied to other targets where elastography is used, such as breast, prostate, and liver.

In this paper, the center frequency is relatively low for monitoring a typical eye. Instead of determining the driving frequency first and selecting the target, we selected the target first that could be easily purchased in large quantities and then selected a driving frequency that could implement the ARFI image of the selected target with our laboratory’s equipment. Although the driving frequency is low, the feasibility of the proposed technique has been successfully verified. Thus, we believe that the performance of the proposed technique can be maintained even if the frequency is increased. In particular, as the frequency increases, the penetration depth decreases, so the effectiveness of the proposed technique with high DOF will be greater. Additionally, in this study, the feasibility of the proposed technique was successfully verified by performing ex vivo experiments, but in vivo imaging experiments should be pursued to further increase the reliability of the performance of the proposed technique. In general, implementing the ARFI image takes more time than the B-mode image. Therefore, the proposed method can be applied to objects with little movement, such as the eye. As the performance of current hardware continues to improve, the time required to implement the proposed method is expected to decrease further.

## 5. Conclusions

In this study, the internal structure of the bovine eye was visualized with biomechanical properties using a phase-inverted ultrasound transducer (PIUT) and the compound acoustic radiation force impulse (CARFI) imaging technique. In the FEA simulation and the experiment using the prototype transducer, respectively, the −3 dB lateral beamwidth of the PIUT driven by the out-of-phase mode increased by 159% and 69% more than that of the in-phase mode. By employing this feature in the pushing beam in ARFI imaging, the lateral beamwidth of the pushing beam can be broader than the detection beam, which is used in-phase mode, and the shearing artifact can be alleviated. In addition, the 60% improved DOF extension effect using the PIUT has been added, allowing a high-resolution far-field image to be obtained via the out-of-phase mode. Furthermore, the CARFI image can provide denser texture in both near-field and far-field regions, showing an about 80% SNR increase compared to the conventional case. Thus, the proposed approach using the PIUT and CARFI imaging techniques can provide high-resolution stiffness information by compounding the in-phase and out-of-phase mode data. Although this study aimed at the crystalline lens, the proposed method can be expanded to monitor ocular diseases accompanied by stiffness change, especially in deeply located regions. In future work, we plan to apply the proposed technique to the array-based ultrasound imaging system for in vivo imaging.

## Figures and Tables

**Table 1:** Mean SNR, Minimum-Maximum SNR, and improvement for each ARFI image mode.

Label *	Imaging Mode	Mean SNR	Min–Max (SNR)	Improvement (%)
(a)	ARFI Image (In-phase)	0.572	0.544–0.604	-
(b)	ARFI Image (Out-of-phase)	0.783	0.762–0.818	36.88
(c)	CARFI Image (In-phase)	0.724	0.678–0.779	26.57
(d)	CARFI Image (Out-of-phase)	0.979	0.922–1.047	71.15
(e)	CARFI Image (In-phase + Out-of-phase)	1.032	1.003–1.059	80.42

* Label indicates each imaging mode.

## Data Availability

Data are contained within the article.
